# One Year of SARS-CoV-2: How Much Has the Virus Changed?

**DOI:** 10.3390/biology10020091

**Published:** 2021-01-26

**Authors:** Santiago Vilar, Daniel G. Isom

**Affiliations:** 1 Department of Molecular and Cellular Pharmacology, University of Miami Miller School of Medicine, Miami, FL 33136, USA; sxv548@med.miami.edu; 2 Sylvester Comprehensive Cancer Center, University of Miami, Miami, FL 33136, USA; 3 Institute for Data Science and Computing, University of Miami, Coral Gables, FL 33146, USA

**Keywords:** SARS-CoV-2, COVID-19, mutations, proteome, sequence, 3D proteins

## Abstract

**Simple Summary:**

Now that vaccines have been developed and are being deployed to address the COVID-19 pandemic, a major concern is the emergence of mutations in severe acute respiratory syndrome coronavirus 2 (SARS-CoV-2) that confer immune escape or enhanced fitness. As such, it is important to assess how rapidly the virus is mutating to gauge the likelihood of such an event. Using ≈290,000 SARS-CoV-2 proteome sequences deposited in a resource known as the Global Initiative on Sharing All Influenza Data (GISAID), we show that 27 of the proteins comprising the SARS-CoV-2 virus are mutating at different rates, with most exhibiting little to no mutational variability. Specifically, we observe that the principal targets of COVID-19 vaccines and therapeutics, the Spike and Nucleocapsid proteins, have the highest mutational variability. Additionally, we provide the foremost assessment of SARS-CoV-2 mutations in terms of time, geography, and their location in the available 3D protein structure. Together, these data demonstrate that the SARS-CoV-2 proteome is slowly accumulating mutations. These finding suggest that extant vaccines and therapies will likely remain effective for the foreseeable future, but the continued surveillance for mutations in primary viral targets is warranted.

**Abstract:**

Severe acute respiratory syndrome coronavirus 2 (SARS-CoV-2) has caused a worldwide crisis with profound effects on both public health and the economy. In order to combat the COVID-19 pandemic, research groups have shared viral genome sequence data through the Global Initiative on Sharing All Influenza Data (GISAID). Over the past year, ≈290,000 full SARS-CoV-2 proteome sequences have been deposited in the GISAID. Here, we used these sequences to assess the rate of nonsynonymous mutants over the entire viral proteome. Our analysis shows that SARS-CoV-2 proteins are mutating at substantially different rates, with most of the viral proteins exhibiting little mutational variability. As anticipated, our calculations capture previously reported mutations that arose in the first months of the pandemic, such as D614G (Spike), P323L (NSP12), and R203K/G204R (Nucleocapsid), but they also identify more recent mutations, such as A222V and L18F (Spike) and A220V (Nucleocapsid), among others. Our comprehensive temporal and geographical analyses show two distinct periods with different proteome mutation rates: December 2019 to July 2020 and August to December 2020. Notably, some mutation rates differ by geography, primarily during the latter half of 2020 in Europe. Furthermore, our structure-based molecular analysis provides an exhaustive assessment of SARS-CoV-2 mutation rates in the context of the current set of 3D structures available for SARS-CoV-2 proteins. This emerging sequence-to-structure insight is beginning to illuminate the site-specific mutational (in)tolerance of SARS-CoV-2 proteins as the virus continues to spread around the globe.

## 1. Introduction

The novel severe acute respiratory syndrome coronavirus 2 (SARS-CoV-2) and the resulting COVID-19 pandemic are causing a global public health and economic crisis [1,2,3,4,5]. Similar to related coronavirus, such as MERS-CoV and SARS-CoV [6,7,8], SARS-CoV-2 has a 29.9 Kb positive-sense single-stranded RNA genome that encodes 29 viral components [7,9]. Most of these components (16 total) are non-structural proteins transcribed as two large polyproteins (Orf1a and Orf1b) that are processed into individual polypeptides by viral proteases (Mpro and PLpro). The remainder of the viral proteome encodes for a variety of accessory and structural components, including the Spike (S), Envelope (E), Membrane (M), and Nucleocapsid (N) proteins.

Mutations provide the virus with mechanisms to increase the transmissibility, modify pathogenicity, and evade host immunity, shifting the antigenic response and causing resistance to therapeutics. SARS-CoV-2 is an RNA virus, which is a family with significant adaptive evolution [10]. Although the changes in coronaviruses are slower than most RNA viruses, there are some viral components in SARS-CoV-2 that already yielded relevant mutations [10,11,12,13,14,15,16,17,18]. In addition, there are differences in the behavior of the multiple viral components. Some proteins, such as the Spike protein, seem more susceptible to mutations, which is likely due to its pivotal role in entering the host cells and altering infectivity. The functional mean and evolutionary importance of most of the SARS-CoV-2 mutations are still being investigated. Moreover, our results indicate that as more data become available, new viral mutations arise, and further monitoring will be necessary to evaluate their role. Continue surveillance and knowledge of the main mutations along with their functional mean can help reduce the healthcare impact, improve response during the pandemic, and contribute to the successful development of effective vaccines and drugs that advance in the clinical process.

Worldwide research groups are generating and sharing SARS-CoV-2 proteome sequence data in a rapid fashion as a global effort to combat the COVID-19 pandemic. The Global Initiative on Sharing All Influenza Data (GISAID) [19] contains more than 250,000 SARS-CoV-2 proteome sequences labeled by date and region. The Protein Data Bank is another crucial resource of viral protein information [20]. Three-dimensional (3D) structures are available for multiple viral proteins, including structural proteins, such as the Spike and Nucleocapsid, the viral proteases Mpro and PLpro, and some non-structural proteins such as NSP12 (RNA-dependent RNA polymerase), NSP15 (Endoribonuclease), or the NSP16–NSP10 complex, among others. A combination of both resources, i.e., mapping sequence data with the available structures from the Protein Data Bank (PDB), provides insights with direct applications in the design of diagnostic tests, vaccines, and drugs. Through this type of analysis, we can also generate hypotheses about the effect(s) of mutations on viral protein function and viral biology.

In this article, we analyze and describe how much the SARS-CoV-2 virus proteome has changed in the first year of the COVID-19 pandemic. Using sequences for ≈290,000 proteomes deposited in GISAID, we quantified the mutations rates for the global SARS-CoV-2 proteome and the individual residues in 27 viral proteins. We performed a dynamic temporal and geographical analysis to monitor the emergence and distribution of these mutations. Additionally, we mapped the mutation rates of individual residues into the current set of 3D structures available for SARS-CoV-2 proteins. As such, the advent of openly available sequence data and improved technologies for rapidly obtaining protein structures have enabled a near real-time assessment of mutations that emerge in the early and middle stages of a viral pandemic.

## 2. Methods

### 2.1. Sequence Data and Residue Mutation Rates

We accessed the GISAID database on 30 December 2020 and downloaded the complete SARS-CoV-2 sequence aligned data from December 2019. Our database was composed of ≈290,000 sequences representing 27 viral proteins. The residue mutation rates (MRs) of the human sequences were calculated in Python [21] considering sequences with the same length, including gaps, as the original Wuhan sequences extracted in December 2019 for all the viral proteins. Residue MRs for protein *j* were computed as the ratio between the frequency in which the original residue is replaced in the protein *j* sequences and the total number of analyzed protein *j* sequences. As an example, the mutation rate for residue *X* in a protein is defined as:

$$\mathrm{MR}\left(X\right)=\frac{\mathrm{Number}\;\mathrm{of}\;\mathrm{sequences}\;\mathrm{with}\;\mathrm{mutated}\;\mathrm{residue}\;\mathrm{in}\;\mathrm{position}\;X}{\mathrm{Total}\;\mathrm{number}\;\mathrm{of}\;\mathrm{sequences}}.$$

**Figure 1:**
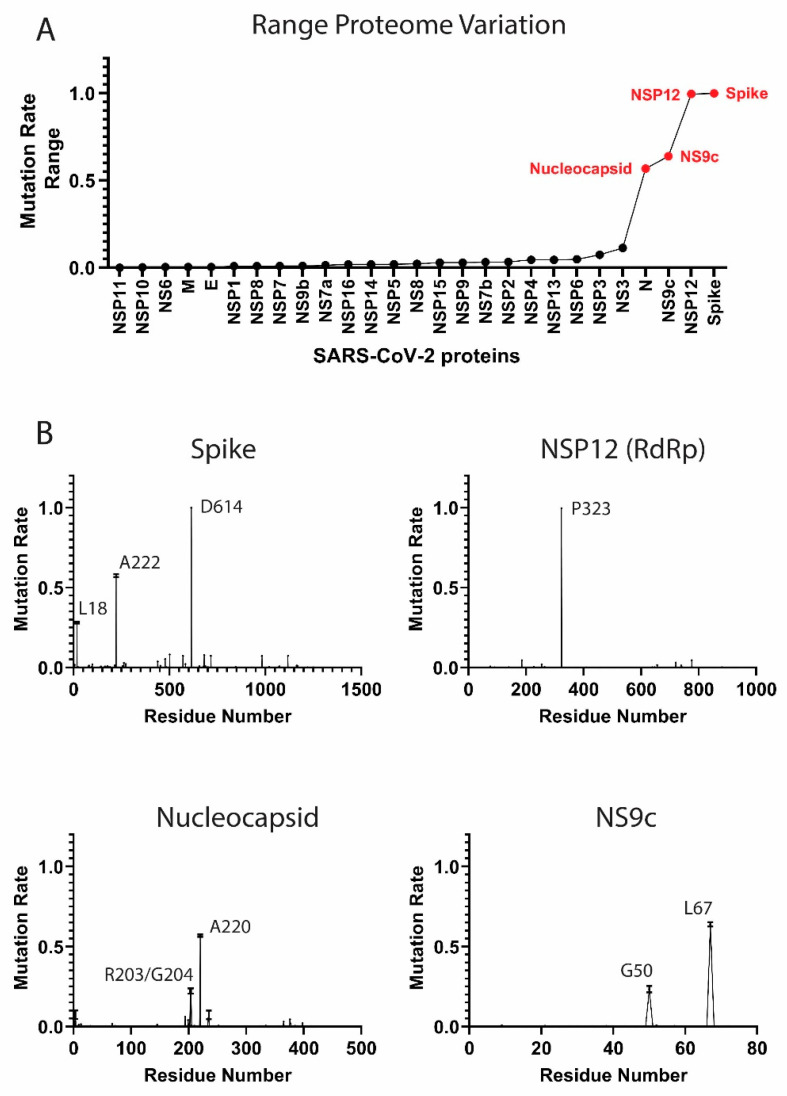
Mutation rates in the severe acute respiratory syndrome coronavirus 2 (SARS-CoV-2) proteome. (**A**) Proteome-wide analysis of the observed mutation rate range for 27 SARS-CoV-2 proteins. Range for each protein is calculated as the difference between the highest residue MR and lowest MR. Red labels correspond to proteins with a range > 0.50. (**B**) Select examples of high-frequency mutating SARS-CoV-2 proteins (main mutation rates in residues D614 (S), A222 (S), L18 (S), P323 (NSP12), R203 (N), G204 (N), A220 (N), G50 (NS9c), and L67 (NS9c). Standard deviation for the mutation rates is plotted). A comprehensive analysis of the mutation rates for the rest of SARS-CoV-2 proteins is available in Figure S1 of the Supplementary Material. MRs were calculated taking into account November–December data.

MRs for Figure 1 were calculated comparing sequences from November to December 2020 against original sequences from China in December 2019. As a measure of statistical error, we calculated the standard deviation associated to each residue MR. We randomly selected 50% of the November–December 2020 sequences and calculated the MRs. This process was repeated ten times. Standard deviation was calculated using the different MR measurements. As a measure of protein variability, we calculated the range for each viral protein. The range is defined as the difference between the highest and lowest values—in our case, the difference between the highest residue MR and the lowest residue MR for each protein (example: the range of the Spike is 1, the difference between the maximum MR (MR of residue D614 is 1) and the minimum MR (value of 0)).

### 2.2. Temporal Analysis

Temporal fragmentation of the data was carried out extracting the sequences labeled according to the date and corresponding to consecutive months. For each period, we performed MRs calculations for each residue in the proteins and the global proteome using Python [21]. Protein variation was computed as the average of all its residue MRs. Periodical proteome variation was calculated as the average of all protein variations in each month. For clarity in the analysis, we considered two periods in the pandemic: a first period from December 2019 to July 2020 and a second period from August to December 2020.

### 2.3. Temporal/Geographical Analysis

Temporal and geographical fragmentation and data analysis were performed in Python and MATLAB [21,22]. Sequence data was partitioned by date and country (≈125 worldwide countries). We manually inspected and unified multiple names representing the same country. Residue MRs were computed as described above and plotted in world maps using MATLAB.

Association analysis between residue mutation rates and mortality in the countries were implemented with April 2020 data (period with high volume of available sequences and peak of the pandemic). We defined positive and negative cases using multiple thresholds for the mutation rates and mortality, which was measured as deaths per million [23]. The analysis calculated an overrepresentation of countries with high/low residue mutation rates and high/low mortality. Enrichment factors with associated *p*-values were computed. All the residues were included in the analysis and *p*-values were corrected by multiple hypothesis using Bonferroni and Benjamini–Hochberg False Discovery Rate (BHFDR) methods.

### 2.4. Protein Structure-Based Mutational Analysis

Mapping sequence mutations into colored 3D crystallized proteins was performed in PyMOL [24]. The set of viral proteins with available crystallized structure in the PDB are part of the analysis. We computed MRs for this section comparing sequence data from November to December 2020 against initial SARS-CoV-2 sequences sent by December 2019.

## 3. Results and Discussion

### 3.1. Components of the SARS-CoV-2 Proteome Are Mutating at Different Rates

The multiple viral components behave in a different manner from a mutational perspective. In this section, we analyzed the components of the SARS-CoV-2 proteome and identified the high frequency mutating viral proteins along with initial and more recent relevant residue mutations.

The SARS-CoV-2 proteome sequences, epidemiological, temporal and geographical data are available at the GISAID initiative [19]. We collected ≈290,000 full SARS-CoV-2 proteome aligned sequences from GISAID along with additional metadata from December 2019 to December 2020. For each viral protein, we calculated individual residue mutation rates (MRs) and ranked residue variability to study the main viral mutations (Figure 1 and Figure S1 of the Supplementary Material. Residue MRs and standard deviations are also provided in Table S1 as part of the Supplementary Material). MRs were calculated comparing November–December 2020 data (latest data) against the initial December 2019 sequences from China. We included both months to provide robustness to our calculation, since we collected ≈42,000 sequences in November 2020 and ≈7000 sequences in December 2020. It is worth noting that GISAID is a retrospective database. Although we downloaded the database on December 30th, more sequences will be sent and collected for December 2020 during the next weeks.

As a measure of protein variability, we calculated the range in the residue mutation rates for each protein in the proteome. The proteins with highest range are the Spike, NSP12, NS9c, and Nucleocapsid (Figure 1A). Our analysis showed that the viral components are evolving at different rates. Some proteins, such as the Envelope (E) protein, have low MRs across the residue sequence, while other viral components, such as the Spike (S) or the Nucleocapsid (N) proteins, showed a higher degree of variability. Our results yielded some residues with higher mutation rates and confirmed some important mutations already described in the bibliography.

#### High-Frequency Mutating SARS-CoV-2 Proteome Components

Several SARS-CoV-2 proteins are mutating at appreciably rapid rates. While it is currently unclear if these mutations benefit the virus, their continued surveillance and the detection of new proteome variants are likely to illuminate key aspects of viral function. As will be discussed, the identification and analysis of mutations in the Spike protein are beginning to provide such insight [11,25]. However, the effects of mutations in other high-frequency mutating viral components, such as the Nucleocapsid and NS9c proteins, is less clear. Here, we summarize the high-frequency mutations that have been observed in the SARS-CoV-2 proteins. MRs were calculated according to the latest data from November to December 2020.

*Spike protein*. The residue D614 of the Spike (S) protein showed a mutation rate of ≈1. The D614G mutation has already been studied in different publications [11,15]. The Spike (S) glycoprotein mediates the entry of SARS-CoV-2 into the host cells. The D614G mutation has been associated with an increase of infectivity but not with an augment of the disease severity [11,25]. A222V and L18F in the Spike were also mutations detected in our analysis (MRs = 0.58 and 0.28 respectively) (Figure 1B). The mutation L18F was also recently detected as part of the variant 501Y.V2 described in South Africa [26]. However, mutations such as E484K or K417N from the variant 501Y.V2 are marginally present in our data (MRs ≈0.004). Other recent mutations from new viral variants, such as the VUI 202012/01 described in the United Kingdom [27], are also detected in our analysis, although the MRs are still low (MRs ≈0.08 for residues N501, A570, P681, T716, S982, and D1118,). We identified a significant increment of the frequency of these mutations when we analyzed only the December data (MR ≈0.27). However, we collected only ≈7000 sequences in our latest December data, and the majority were sent from the United Kingdom. Further surveillance is a crucial step to control the evolution and estimate the expansion of the variant.

*NSP12 protein*. The P323L mutation in the NSP12 (RNA-dependent RNA polymerase, RdRp) protein accompanies the D614G (S) mutation in most of the analyzed sequences (MR = 0.996). This dual mutation has also been reported for multiple research groups [11,28]. As RdRp catalyzes the replication of RNA, the P323L mutation could affect the speed of the viral replication [28]. However, the P323L mutation is situated far away from the catalytic site. Other mutated residues in RdRp showed lower mutation rates, such as A185 (MR = 0.046) and V776 (MR = 0.046) and occupied remote positions from the pocket.

*Nucleocapsid protein*. The Nucleocapsid is another target essential in the production of viral particles, which is involved in RNA replication, transcription, and genome assembly [29]. The Nucleocapsid also presented two consecutive residues with high mutation rates, which were equivalent to the mutations R203K and G204R (MRs = 0.22) (Figure 1B) [15]. Although these mutations generated lower expectation in previous literature, the residues could impact key regions for the transcription and replication of SARS-CoV-2 [30]. However, our latest data indicate that the virus is mutating back to its initial form in those residue positions. Another mutation in the Nucleocapsid, the A220V, has gained importance recently (MR = 0.57). This mutation along with A222V (Spike) have been already included in a viral variant spread in Europe during the summer 2020 (variant 20A.EU1) [31]. The next most mutated residues in the N protein were D3 (MR = 0.07), S235 (MR = 0.07), S194 (MR = 0.06), and M234 (MR = 0.05). Further monitoring of D3 and S235 is important, since the MRs of those residues increased considerably in the latest data from December (MR ≈0.27).

*NS9c accessory protein*. Mutations such as G50N (MR = 0.23) and L67F (MR = 0.64) in the NS9c are highly correlated with residues R203/G204 and A220 from the Nucleocapsid due to possible overlapping in the reading frame.

Other viral proteins showed mutations in multiple positions, although the mutation rates are notably lower (Figure S1). Changes in residue Q57 in the NS3 (MR = 0.11) [32], or residues T183 (MR = 0.08), I1412 (MR = 0.07), and A890 (MR = 0.07) in the NSP3 should also be further monitored (MRs for the cited residues in NSP3 increased to ≈0.27 in December). More studies would be necessary to clarify their implication in the viral cycle life.

### 3.2. The Temporal Emergence of Proteome Mutations

As coronaviruses have high adaptive evolution, we expect that SARS-CoV-2 presents significant temporal variations. Some factors can condition the different viral variants. Growing evidence indicates that climate and seasonal effects, including temperature, humidity, sunlight, and people’s habits, can contribute to the expansion of the virus [33]. Country-specific factors, such as demography, cultural practices, social interventions, travel restrictions, quarantine policies, health care capacity, and reporting and tracking mechanisms, can also alter viral expansion and variation. We analyzed the evolution of the SARS-CoV-2 virus over a year, including multiple seasons. As expected, temporal analysis yielded important variations in short periods of time. Here, we provide multiple examples of temporal differences in viral protein mutation rates that exhibit a variety of behaviors.

**Figure 2:**
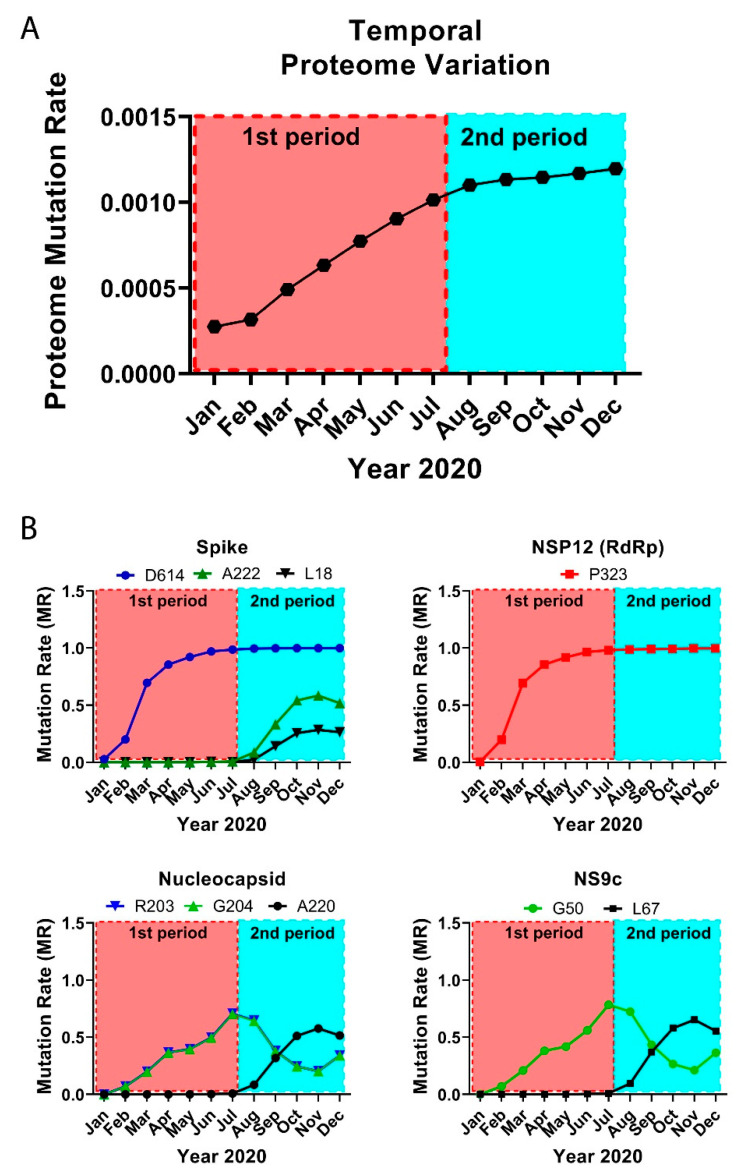
Temporal emergence of SARS-CoV-2 mutations. (**A**) Running temporal average of SARS-CoV-2 proteome variation relative to December 2019. (**B**) Select temporal counts of SARS-CoV-2 variation rates for the high frequency mutating residues in the Spike, NSP12, Nucleocapsid, and NS9c proteins.

We divided the GISAID global sequence data over several months and performed a temporal residue mutation analysis for the whole proteome and the main mutations D614G (S), A222V (S), L18F (S), P323L (NSP12), R203K (N), G204R (N), A220V (N), G50N (NS9c), and L67F (NS9c) (see Figure 2). MRs in this section were computed comparing the sequences from each consecutive month against initial December 2019 data. Global analysis of the proteome temporal data showed two different periods in the mutational evolution of SARS-CoV-2. We observed two different mutational tendencies from December 2019 to July 2020 (first period) and from August to December 2020 (second period). The global results were confirmed by the detailed temporal analysis of the individual residue mutations.

The virus proteome changed gradually over time, reaching a maximum variation in the last analyzed month, December 2020 (global proteome MR = 0.0012). However, the temporal analysis showed two periods with different slope in the mutational variation: in the first period, until July 2020, the proteome changed more abruptly, and the mutation rate rose rapidly, whereas the second period, from August until December 2020, showed a proteome stabilization with a slight increase in the global mutation rate (Figure 2A). Both periods are more remarkable when we analyze from a temporal perspective the main residue mutations.

When we investigated the residue mutations occurred during the first period, such as the D614G and P323L mutations, the ascend in the residue mutation rate is steeper in March with an abrupt rise in the mutation rate from 0.20 to 0.69 until a current value of ≈1 (Figure 2B). It is worth noting that the sequences collected from March to April represented ≈30% of the complete dataset. The mutation rates for residues R203/G204 of the Nucleocapsid and G50 in NS9c increased in a more gradual fashion until reaching values of ≈0.70–0.78 in July 2020. However, from that date onwards (second period), the MR in those residues decreased to a value of ≈0.22 in November 2020. The MRs raised slightly in December 2020 (≈0.35).

In our data, there is a group of residues that played a more important role during the second period. The MRs of residues A222 (S), A220 (N), and L67 (NS9c) started to gradually increase in August until yielding values of ≈0.58–0.65 in November 2020. The MRs detected in December 2020 for residues A222 (S), A220 (N), and L67 (NS9c) changed the tendency and decreased slightly. L18 (S) followed a similar pattern as A222 (S) and reached an up-to-date MR of 0.26. These new mutations should be further monitored to establish if they play a key role in the viral life cycle.

It is worth mentioning that in the latest period, December 2020, new mutations were identified in the Spike, Nucleocapsid, and NSP3 proteins. Mutations in different residues of the Spike (variant VUI 202012/01) presented an MR of ≈0.27 in December 2020. Similar MRs (≈0.27) were obtained for D3 and S235 of the Nucleocapsid and T183, A890, and I1412 of the NSP3. The new mutations and the change in the MR tendency detected in December 2020 for some residues in the Spike, Nucleocapsid, and NS9c (Figure 2B) could mean that we are starting a third mutational period. However, our December data contain only ≈7000 sequences, whereas other months, such as November 2020 cover ≈42,000 sequences. In addition, the majority of the sequences in the last months were sent from the United Kingdom. It is important to further monitor the changes in those residues to estimate the expansion and impact of the mutations.

### 3.3. Worldwide Geographical and Temporal Differences in Proteome Variation

**Figure 3:**
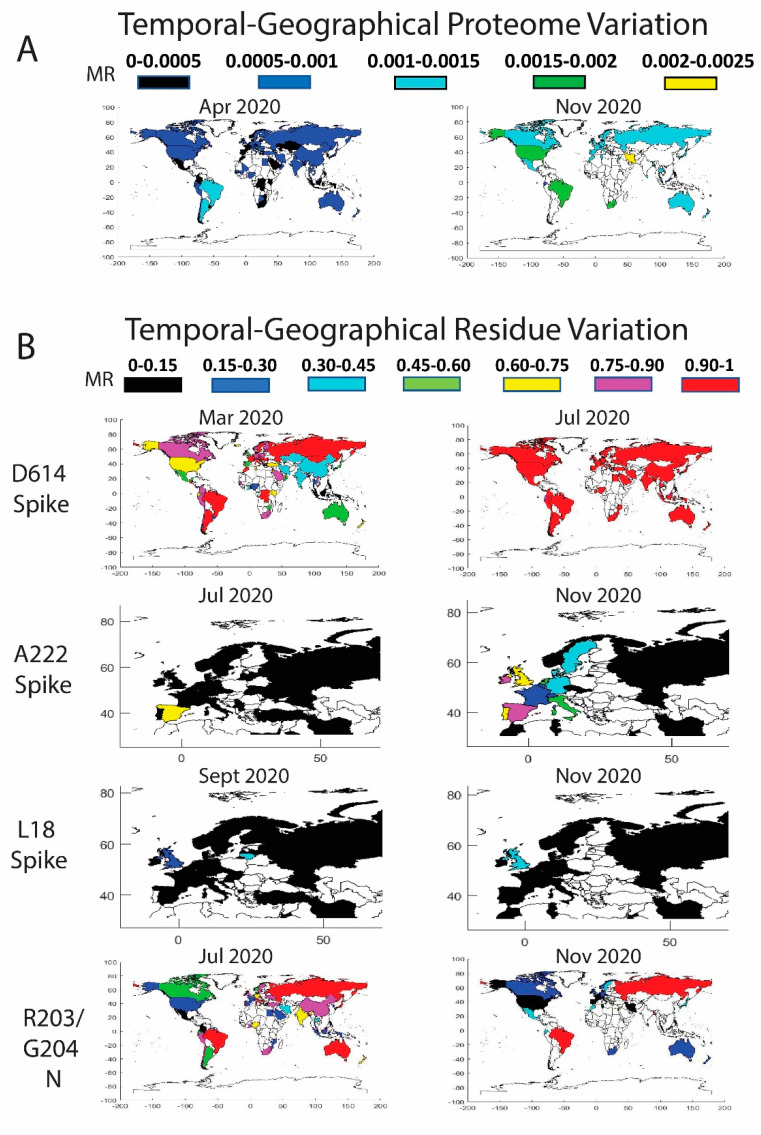
Temporal worldwide mutation rate (MR) analysis for the complete SARS-CoV-2 proteome (**A**) and the high-frequency mutating residues (**B**): D614 in the Spike (correlated data for P323 in the NSP12), A222 in the Spike (correlated data for A220 in the Nucleocapsid and for L67 in the NS9c), L18 in the Spike, and R203/G204 in the Nucleocapsid (correlated data for G50 in the NS9c). A minimum threshold of five sequences was considered in the world plots.

As described previously, country-specific factors contribute to the viral variation and generate different patterns in the pandemic expansion. Our analysis indicated geographical differences in viral protein mutation rates and exhibited a variety of expansion behaviors. From a global perspective, we detected progressive increments in the proteome variability by country throughout the 2020. In agreement with Figure 2A, the proteome in April presents an average MR between 0.0005 and 0.001 in multiple countries worldwide (Figure 3A). The proteome MR increases during the second period and overcomes the 0.001 threshold in most of the countries in July–August 2020.

We also monitored the residue mutation rate over time in different geographic regions of the world for residues D614 (S), A222 (S), L18 (S), P323 (NSP12), R203 (N), G204 (N), A220 (N), G50 (NS9c), and L67 (NS9c) (Figure 3B). A detailed description has been included in Tables S2–S5 of the Supplementary Material with the date, country, MRs, and number of sequences for the main residues described in Figure 3. Our data showed that D614G (S) and P323L (NSP12) mutations overtook the entire globe. Mutations R203K (N), G204R (N), and G50N (NS9c) spread over the world but are less stable than the mutation D614G (S), and those residue positions were subjected to back-mutation toward the original state in multiple areas. More recent viral mutations, such as A222V (S), L18F (S), A220V (N), and L67F (NS9c) were mostly detected in Europe and should be further monitored to estimate their impact in viral evolution. A detailed description of the geographical evolution of these mutations is provided below.

*Spike.* The D614G mutation was already present in January 2020 in the sequences analyzed from Germany (MR = 1, sequences = 9). We detected in that period the D614G mutation in Australia and China, but the original residue was still highly conserved (MRs = 0.05 and 0.01 respectively). Surprisingly, the sequences evaluated from Germany in February showed a decrease in D614 mutation (MR = 0.45). As previously reported [11], half of the analyzed sequences coincided with the initial Wuhan form. However, in February, other countries showed a remarkable increase in the presence of the D614G mutation, such as Saudi Arabia (MR = 1), Switzerland (MR = 0.97), Italy (MR = 0.96), France (MR = 0.78), Austria (MR = 0.75), the Netherlands (MR = 0.63), and Brazil (MR = 0.6 but only five analyzed sequences). The United Kingdom and Spain showed MRs of 0.40 and 0.30. In North America, the U.S. still presented a MR for D614 of 0.07, whereas Canada showed a higher evolution in this period (MR = 0.33). In China, the MR was in the same range as previously reported in January (MR = 0.01). There are additional countries with high D614 MRs, but more representative data would be necessary to extract any conclusions (less than five collected sequences).

As described in Figure 2, the high increase in the incidence of the D614G mutation happened in March, where there are many countries in different areas of the globe with MR higher than 0.90, such as Estonia (MR = 1), Morocco (MR = 1), Argentina (MR = 1), Romania (MR = 1), Faroe Islands (MR = 1), Mongolia (MR = 1), Italy (MR = 0.99), Hungary (MR = 0.98), Bosnia and Herzegovina (MR = 0.96), Russia (MR = 0.96), Switzerland (MR = 0.95), France (MR = 0.94), Croatia (MR = 0.94), Brazil (MR = 0.94), Denmark (MR = 0.93), Luxembourg (MR = 0.93), Czech Republic (MR = 0.93), Costa Rica (MR = 0.92), Sweden (MR = 0.92), and the Democratic Republic of the Congo (MR = 0.91). It is worth mentioning that residue D614 showed a slower evolution in China and neighboring countries in Asia compared to the rest of the world. This situation is remarkable in April 2020, when the mutation rates for the residue were higher than 0.75 in most of the world except in some Asian countries with mutation rates between 0.3 and 0.75. After May 2020, D614 was more than 90% mutated in practically all the globe and the latest data from November to December 2020 show the G614 mutated residue in practically the 100% of the sequences. Based on the difference in the temporal and geographical expansion of the mutation, we performed an enrichment analysis during April 2020 to investigate if there is an association between low D614 mutation rates and reduced mortality (number of deaths per million) in the different countries. Our goal was to investigate if the mutation could cause higher infectivity and, hence, an increase in mortality. Previous studies have shown significant correlations between the presence of D614G mutation and increased case fatality rates [34,35]. We established different thresholds for the MRs and mortality. We detected an enrichment factor > 1.25 with associated *p*-values < 0.05 in six out of 12 calculations. Conversely, we only found significant results in one out of 12 thresholds when we looked for an association between higher MRs or the presence of the D614G mutation and increased mortality. In addition, when we extended our analysis to all the residues in all the proteins (≈10,000 residues), we did not find associations between MRs and mortality. We corrected our analysis by multiple hypothesis using Bonferroni and BHFDR methods [36], and all the possible associations failed the test. More studies are necessary to prove possible associations between SARS-CoV-2 mutations and mortality.

The sequences deposited from July–August to December 2020 yielded new mutations in the SARS-CoV-2 (Figure 3B). According to our data, the A222V (Spike) mutation was already detected in March in Tunisia and Iran, in April in Turkey, and in May in Mexico and Canada, among others, although the MR of the A222 residue was still low (≈0.03). However, in June 2020, the mutation is clearly detected in Spain (MR = 0.43) and mildly in Senegal (MR = 0.05). The mutation spread in July to Gibraltar (MR = 0.2) and slightly to Norway, Belgium, Ireland, and Switzerland (MRs ≈0.06–0.02). The variant with A222V completely overtook Spain in August (MR = 0.84) and continued its expansion to Norway (MR = 0.39), Latvia (MR = 0.24), Switzerland (MR = 0.22), the United Kingdom (MR = 0.17), Denmark (MR = 0.17), Italy (MR = 0.11), and other European countries (France, the Netherlands, Ireland, Sweden, Germany, and Belgium). Outside Europe, the mutation was detected in China although with low rates (MR = 0.05). The data in September showed that the mutation was present mainly in Spain (MR = 0.82), Ireland (MR = 0.51), the United Kingdom (MR = 0.46), Lithuania (MR = 0.44), Denmark (MR = 0.35), Switzerland (MR = 0.34), the Netherlands (MR = 0.33), Germany (MR = 0.21), Belgium (MR = 0.15), Sweden (MR = 0.14), France (MR = 0.13), and Italy (MR = 0.13). The sequences in October–December yielded an increase of the A222V mutation in multiple countries in Europe, in New Zealand (MR = 0.32 in December), and Tunisia (MR = 0.11 in November). A similar distribution pattern was found for the A220V mutation of the Nucleocapsid. Previous studies already confirmed a cluster variant with both A222V and A220V that emerged during the summer, presumably in Spain, and posteriorly spread in Europe [31].

The mutation L18F in the Spike was marginally present in the United Kingdom in February and in different countries in March (MRs ≈0.005). The data showed that the mutation was residually present in multiple countries until it expanded into the United Kingdom (MR = 0.07 and 6798 analyzed sequences), China (MR = 0.05, 44 sequences), and Colombia (MR = 0.13 but only eight analyzed sequences) in August 2020. We detected in September an increase in the incidence of the mutation in Lithuania (MR = 0.4, 25 sequences), the United Kingdom (MR = 0.23 and 14,968 sequences), Chile (MR = 0.2, only five available sequences), Ecuador (MR = 0.11, nine sequences), Ireland (MR = 0.07, 182 sequences), Germany (MR = 0.04, 130 sequences), Sweden (MR = 0.03, 66 sequences), Singapore (MR = 0.03, 33 sequences), and Latvia (MR = 0.03, 37 sequences). The data in November 2020 showed a MR in the United Kingdom of 0.39 (29,953 sequences) and 0.14 in Ireland (102 sequences) (Figure 3B). L18F is a mutation also included in the variant 501Y.V2 [26]. Future surveillance of the new Spike mutations is necessary to estimate the importance of the variations.

*NSP12*. The viral variant with D614G contains also the P323L mutation in the NSP12. As a result, same conclusions can be extracted for both variations. We observed a clear correlated evolution by country between residues D614 and P323 (Figure 3B).

*Nucleocapsid*. The mutations in the Nucleocapsid, which are located mainly in residues R203 and G204, showed different evolution patterns compared to D614G (Figure 3B). In February 2020, different European countries already displayed the R203K mutation. The residue was highly mutated in the sequences analyzed from Switzerland (MR = 0.76), Austria (MR = 0.75), and the Netherlands (MR = 0.56), although more countries exhibited the mutation with lower mutation rates, such as Italy (MR = 0.20), Germany (MR = 0.17), France (MR = 0.13), Spain (MR = 0.13), and the United Kingdom (MR = 0.13). In this period, the mutation was incipient in U.S. (MR ≈0.04). In March 2020, the R203K mutation had already extended to other countries, such as Brazil, Greece, the Czech Republic, Estonia, Ireland, Russia, and Vietnam, among others, with an MR higher than 0.5. Nevertheless, it was in Japan, Brazil, and Vietnam in April and in Lithuania, Russia, Oman, and Zimbabwe in May, where the R203K mutation reached the threshold of 90%. The residue evolution in the U.S. was slower, but in May 2020, the mutation rate increased to 0.15. The rate increased again in June until 0.22, although the data in July showed contradictory conclusions with a lower MR of 0.18. The MR continued to decline until reaching a value of 0.06 in December. The MR decrease in the U.S. was not an isolated phenomenon, and the virus after July 2020 retrieved the primitive residue in multiple countries. A similar pattern was found for residue G204 (N) with a decline in the MR in the last months in most of the countries. We cannot determine the cause of the R203/G204 back-mutations. Reversion of the mutational process to the original residue is part of viral evolution and MR dynamics [37]. As a hypothesis, viral variants with additional mutations but with the original R203/G204 residues could have increased their frequency and expansion around the globe due to higher infectivity and so diminish the percentage of the viruses containing the R203K/G204R mutations. As described previously, the evolution of residue A220 in the Nucleocapsid is highly correlated with the data obtained for residue A222 in the Spike.

*NS9c*. Residues L67 and G50 in the NS9c showed similar expansion patterns as residues A220 and R203/G204 in the Nucleocapsid. Overlapping in the reading frame could be the cause of the highly correlated evolution detected for these residues.

### 3.4. Residue Variation at 3D Molecular Level: Mapping into Crystallized Proteins

**Figure 4:**
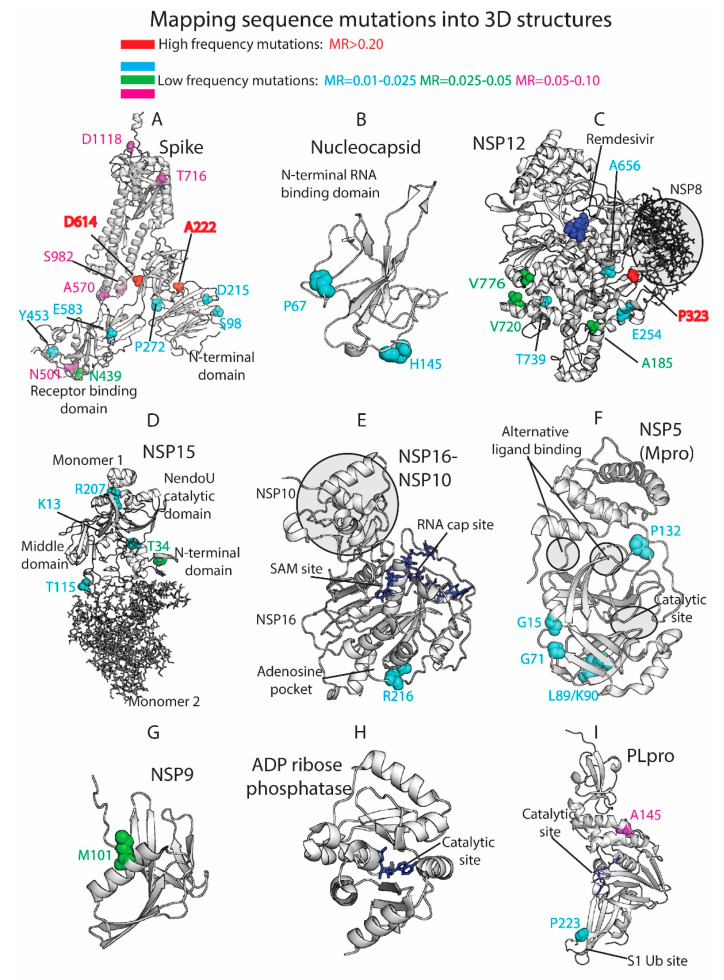
Three-dimensional (3D) protein structures colored by residue mutation rates: Spike, Nucleocapsid, RdRp (NSP12), Endoribonuclease (NSP15), NSP16-NSP10 heterodimer, Mpro (NSP5), NSP9, ADP ribose phosphatase (NSP3), and Papain-like protease (PLpro, NSP3). Proteins represented in white ribbons (MRs < 0.01) and color-coded residues (cyan: MRs = 0.01–0.025, green: MRs = 0.025–0.05, magenta: MRs = 0.05–0.10, red: MRs > 0.20. No residues with MR values between 0.10 and 0.20 were available in the shown crystallized structures).

The 3D analysis of the viral mutations contributes to understanding the key role of specific residues, helps in the assessment of pharmacological targets, and guides the design and development of novel therapeutics. We mapped the SARS-CoV-2 sequence mutations into the crystallized 3D protein structures available in the Protein Data Bank (PDB) [20]. We plotted high-frequency mutations (already described throughout the manuscript) and low-frequency mutations. Most of the proteins are highly conserved, and the punctual mutations are not close to the main catalytic sites. Multiple viral proteins could be promising drug targets from the evolutionary perspective. Figure 4 shows the main mutations located in the 3D SARS-CoV-2 protein structures.

The Spike (S) is a homo-trimeric transmembrane glycoprotein that mediates the viral entry into the host cells [38,39,40]. The Spike is the main target in the development of most of the vaccines [41] and residue variability could affect protective efficacy. The protein contains two subunits, S1 (14–685 residues) and S2 (686–1273 residues), in charge of binding to the host receptor and fusion of the host and viral membranes. The main mutation located at D614 is on the surface of each protomer (Figure 4A). The D614 established a stabilizing hydrogen bond with the residue T859 of the adjacent protomer. The mutation D614G could interrupt the mentioned hydrogen bond between both protomers, provide higher protein flexibility, or even modify glycosylation at close residues, such as N616 [11]. As we have shown in our prior work [42,43,44,45], ionizable residues can be important for the pH responses of proteins, including viral components. Given the influence of pH in viral entry mechanisms [46] and the nature of D614 as an ionizable residue, the mutation could affect the pH-dependent responsiveness of the virus as it enters through the increasingly acidified endocytic pathway. One motivation for our efforts in the future will be to assess the frequency with which ionizable residues (E, C, D, H, K, R, Y) are mutated in viruses, such as SARS-CoV-2, and their role in pH-dependent endocytic entry.

The mutations A222V and L18F are far from the main D614G mutation and are located in the N-terminal domain of the S1 subunit. Alanine substitution by the bulkier valine in A222V can change inter-residue contacts and the 3D structure of the region. Both mutations D614G and A222V are located within areas defined as possible B-cell epitopes [47]. This could provide to the virus an evasive immunological advantage to avoid B-cell response. L18F is not represented in the 3D structure, since the crystallized protein is missing residues M1-P26. The crystallized structure is also missing residues P681 (MR = 0.08), S477 (MR = 0.05), and A262 (MR = 0.03), among others. Most of the mutations that define the UK variant are represented in Figure 4A, such as N501, A570, T716, S982, and D1118. Additional mutations with lower mutational rate are also represented, such as S98, D215, and P272 in the N-terminal. N439 and Y453, along with the cited residue N501, are located in the Receptor-Binding Domain (RBD), which is an essential region in the binding of the host cell receptor ACE2.

Moreover, a former study in SARS-CoV associated residues 1–422 of S1 with the induction of COX-2 expression [48]. Although additional studies in SARS-CoV-2 would be necessary, mutations in this area of the S protein in the SARS-CoV-2 could be implicated in COX-2 expression and related to the inflammation response and severity of the disease. In addition, mutations can have an impact on protein stability [49]. The Spike protein from SARS-CoV-2 gained stability compared to SARS-CoV [50], and this fact could be important in the increasing spread of the virus. Mutations in the Spike could contribute to the stability of the protein and, hence, the viral entry and propagation.

The Nucleocapsid participates on the vital cycle of the virus in RNA assembly and release of viral particles [51]. It is an important target for pharmacological intervention not only in the discovery of drugs but also in the development of vaccines [52]. Some of the residue mutations could interfere in the pharmacological intervention. The SARS-CoV-2 crystallized structures available at the PDB show the N2b and the RNA binding domains and do not contain key residues from the evolutionary perspective (Figure 4B). Residues R203/G204 (MR ≈0.22) are not present in the crystallized structures along with A220 (MR = 0.57), D3 (MR = 0.07), S235 (MR = 0.07), S194 (MR = 0.06), M234 (MR = 0.05), and A376 (MR = 0.04), among other low-frequency MR residues.

The NSP12 protein, also called RNA-dependent RNA polymerase (RdRp) is an important pharmacological target in viral intervention. Mutations in different viral RdRPs have been associated with drug resistance [53,54]. The FDA-approved treatment for COVID-19, Remdesivir, binds the catalytic site of RdRp, causing a decrease in the production of viral RNA. Our analysis showed that RdRp in SARS-CoV-2 is highly conserved as 924 residues out of 932 yielded MRs < 0.005 (see Figure 4C). The residues with higher MRs, P323 (MR = 0.996), V776 (MR = 0.046), A185 (MR = 0.046), V720 (MR = 0.03), E254 (MR = 0.02), A656 (MR = 0.016), and T739 (MR = 0.012), are not close to the catalytic site. However, computational studies have shown that P323L and A185V mutations could have an effect in the preservation of the secondary structure of the protein that could affect protein function and drug binding [55]. Alternatively, a possible binding site was described in a hydrophobic region in close proximity to P323 [14]. RdRp forms a polymerase complex with NSP7 and NSP8 to improve RNA synthesizing activity. This complex can associate with NSP14, which is involved in replication fidelity [56]. Mutations that alter complex interactions could affect RNA replication. In fact, the mutation P323L is near the binding region between NSP12 and NSP8 and could have an impact in the polymerase complex stability (Figure 4C).

NSP15, the viral endoribonuclease, is another possible drug target that was analyzed from the mutational point of view. The main mutated residues are T34 (MR = 0.03), K13 (MR = 0.01), R207 (MR = 0.01), and T115 (MR = 0.01) (Figure 4D). The protein is highly conserved, and no important mutations were detected close to the catalytic site. However, some of the cited residues could collaborate in the formation of the oligomeric structure. The protein is a hexamer where the different monomers interact each other. The assembly of the hexamer is potentially sensitive to the mutations, especially in the N-terminal and middle domains [57]. T34, located in the N-terminal and T115 located in the middle domain could play a role in the stabilization/destabilization of the hexamer with important implications for the Endoribonuclease functionality.

The heterodimer NSP16-NSP10 protects SARS-CoV-2 from the host immune response [58]. Additionally, the disruption of NSP16 decreased the production of RNA in SARS-CoV [59]. Targeting NSP16 can facilitate immune response and decrease pathogenicity and, hence, it could be a key target in drug design. Furthermore, multiple binding sites have been described [58], including the S-adenosyl methionine (SAM) site, the RNA cap substrate cavity, and a third distant pocket unique to SARS-CoV-2 bound to adenosine. Our sequence analysis showed low mutation rates for residues in both NSP16 and NSP10. From a mutational perspective, the NSP16 pockets are highly conserved and composed of residues with MRs lower than 0.01 (Figure 4E). Residue R216 (MR = 0.02) is close to the adenosine-binding pocket. The important functionality of the NSP16–NSP10 complex, diversity in the binding sites, and mutational stability point to the heterodimer as an interesting drug target.

Another target studied by multiple research groups from the point of view of drug discovery and design is the viral main protease Mpro (NSP5) [60,61,62,63]. However, Mpro as a promising target for drug discovery against SARS-CoV-2 has raised some concerns [60]. A flexible loop constituted by residues C44-P52 can occlude the accessibility of the catalytic pocket and limit the entrance of the ligands [60]. Additionally, the plasticity of the catalytic site could make it vulnerable even to distant mutations. Our analysis identified low-frequency mutations in K90 (MR = 0.02), L89 (MR = 0.02), G15 (MR = 0.01), G71 (MR = 0.01), and P132 (MR = 0.01) (Figure 4F). The cited residues are not in close proximity to either the catalytic site or two alternative binding areas described in crystallized Mpro structures (PDB_code: 5RFA, 5RGQ, 5RF0). The results showed that the main protease is a very conserved protein with high interest in drug discovery.

Other possible viral pharmacological targets yielded a high degree of conservation in all the residues, such as the RNA replicase (NSP9) with a role in viral RNA synthesis and viral replication [64] (all residues with MR < 0.005 except M101 with MR of 0.03), the ADP ribose phosphatase, unit of the large multidomain NSP3 with possible functionality in the interference of the host immunological response [65] (MRs < 0.01 except H295 with MR of 0.02), and the PL protease, unit of the NSP3 (Figure 4G–I). All the PL protease residues presented MRs < 0.01 except A145 (residue A890 of the NSP3) and P223 (residue P968 of the NSP3) with MRs of 0.07 and 0.02, respectively. The mutation in P223 is in the S1 ubiquitin region, which is one of the binding sites for ubiquitin and ubiquitin-like protein ISG15. This enzyme plays an essential role in the replication and processing of viral proteins [66] but also could decrease host immunological response by collaborating in deubiquitinating and deISGylating activities [67,68]. SARS-CoV-2-PLpro could be an excellent drug target with high residue conservation that participates in viral replication and modulates signaling in infected cells.

### 3.5. Limitations

Low mutation rates and their important role in the virus life cycle make the different viral proteins attractive targets for pharmacological intervention. Although vaccines and therapies could remain effective for the foreseeable future, continuous surveillance is mandatory, especially in the target proteins used in the therapies. The Spike (S) and the Nucleocapsid (N) are the most used proteins in the development of vaccines and constitute also important targets in drug discovery. According to our data, the S and N proteins showed a higher degree of variability in specific residues, and this is a motive of concern in the efficacy of vaccines and therapeutics. Additionally, the accuracy of multiple diagnostic tests could be affected by mutations in both proteins. Many COVID-19 RT-PCR and rapid antigenic tests are based on the detection of specific regions in the N gen [69]. Although most of the PCR assays use multiple targets, mutations in N and S proteins could be involved in the performance of some diagnostic tests, causing an additive burden to the health system.

In our study, we used a representative sequence database provided by the GISAID initiative. However, conclusions about the data could be biased by the different number of sequences sent from multiple areas of the world. Moreover, the data is retrospective, and an important percentage of the sequences for the latest period will be sent and collected during the next weeks. We reported the main SARS-CoV-2 mutations as isolated phenomena, and no cluster mutations belonging to the different variants were studied.

## 4. Conclusions

In this article, we describe a wide and global analysis of ≈290,000 full SARS-CoV-2 proteome sequences from GISAID. We calculated residue mutation rates (MRs) across the whole proteome. We analyzed the mutational landscape from different perspectives considering temporal, geographical, and molecular levels. Our analysis identified two periods with a different mutational landscape, from December 2019 to July 2020, and from August to December 2020. The first period was critical for some previously described mutations that overtook the entire globe, such as the D614G and P323L in the Spike and NSP12, respectively. In the second period, additional mutations in the Spike and the Nucleocapsid were notably detectable in multiple countries, mainly in Europe. The latest data yielded new current mutations that should be further monitored. Our analysis provides new insights about current mutations in the SARS-CoV-2 virus, helps to understand the evolution and expansion of the virus, and facilitates the design of diagnostic tests, vaccines, and drugs.

## Supplementary Materials

The following are available online at https://www.mdpi.com/2079-7737/10/2/91/s1, Table S1. Residue mutation rates (MRs) with values ≥ 0.01 for the SARS-CoV-2 proteome. Sequences from November to December 2020 were compared against the initial sequences from China in December 2019. Tables S2–S5. Mutation rates and number of sequences over time in different geographic regions of the world for residues D614 (S), A222 (S), L18 (S) and R203 (N). Figure S1. Residue mutation rates for the following SARS-CoV-2 proteins: NSP1, NSP2, NSP3, NSP4, NSP5 (Mpro), NSP6, NSP7, NSP8, NSP9, NSP10, NSP11, NSP13, NSP14, NSP15, NSP16, NS3, NS6, NS7a, NS7b, NS8, NS9b, Envelope (E) and Membrane (M).

## Abbreviations

MR	Mutation rate
S	Spike
N	Nucleocapsid
E	Envelope
M	Membrane
RdRp	RNA-dependent RNA polymerase
PLpro	Papain-like protease
Mpro	Main protease
