# Supplementary material for: One Year of SARS-CoV-2: How Much Has the Virus Changed?
Source: Biology (Basel). 2021 Jan 26;10(2):91. doi: 10.3390/biology10020091 (PMC7911924; doi:10.3390/biology10020091)

**Supplementary Material**

**One Year of SARS-CoV-2: How Much Has the Virus Changed?**

Santiago Vilar ^a^ and Daniel G. Isom ^a,b,c,*^

^a^ Department of Molecular and Cellular Pharmacology, University of Miami Miller School of Medicine, Miami, FL 33136, USA

^b^ University of Miami Sylvester Comprehensive Cancer Center, Miami, FL 33136, USA

^c^ Center for Computational Sciences, University of Miami, Coral Gables, FL 33146, USA

* To whom correspondence may be addressed. Email: disom@miami.edu

**Index:**

**Table S1.** Residue mutation rates (MRs) with values ≥0.01 for the SARS-CoV-2 proteome. Sequences from November-December 2020 were compared against the initial sequences from China in December 2019 (SD=Standard Deviation).

**Figure S1.** Residue mutation rates for the following SARS-CoV-2 highly conserved proteins: NSP1, NSP2, NSP3, NSP4, NSP5 (Mpro), NSP6, NSP7, NSP8, NSP9, NSP10, NSP11, NSP13, NSP14, NSP15, NSP16, NS3, NS6, NS7a, NS7b, NS8, NS9b, Envelope (E) and Membrane (M).

**Table S1.** Residue mutation rates (MRs) with values ≥0.01 for the SARS-CoV-2 proteome. Sequences from November-December 2020 were compared against the initial sequences from China in December 2019 (SD=Standard Deviation).

| Protein | Residue | Number | MR | SD |  | Protein | Residue | Number | MR | SD |
| --- | --- | --- | --- | --- | --- | --- | --- | --- | --- | --- |
| Spike | D | 614 | 1.000 | 0.0002 |  | **NSP6** | A | 54 | 0.022 | 0.0007 |
| NSP12 | P | 323 | 0.996 | 0.0001 |  | **NS8** | A | 65 | 0.021 | 0.0004 |
| NS9c | L | 67 | 0.639 | 0.0130 |  | **Spike** | P | 272 | 0.021 | 0.0004 |
| Spike | A | 222 | 0.575 | 0.0097 |  | **Spike** | E | 583 | 0.021 | 0.0002 |
| N | A | 220 | 0.569 | 0.0070 |  | **NSP2** | I | 120 | 0.021 | 0.0014 |
| Spike | L | 18 | 0.281 | 0.0062 |  | **Spike** | S | 98 | 0.021 | 0.0006 |
| NS9c | G | 50 | 0.233 | 0.0199 |  | **N** | A | 398 | 0.020 | 0.0016 |
| N | R | 203 | 0.223 | 0.0173 |  | **NSP5** | K | 90 | 0.020 | 0.0010 |
| N | G | 204 | 0.222 | 0.0174 |  | **NSP3** | H | 295 | 0.020 | 0.0005 |
| NS3 | Q | 57 | 0.114 | 0.0064 |  | **NSP3** | P | 968 | 0.020 | 0.0014 |
| Spike | N | 501 | 0.081 | 0.0005 |  | **NSP12** | E | 254 | 0.020 | 0.0015 |
| Spike | P | 681 | 0.077 | 0.0003 |  | **NSP14** | M | 501 | 0.020 | 0.0015 |
| NSP3 | T | 183 | 0.075 | 0.0276 |  | **NS3** | Q | 38 | 0.019 | 0.0007 |
| N | D | 3 | 0.074 | 0.0272 |  | **NSP16** | R | 216 | 0.019 | 0.0026 |
| NSP3 | I | 1412 | 0.074 | 0.0277 |  | **NSP5** | L | 89 | 0.018 | 0.0029 |
| N | S | 235 | 0.073 | 0.0270 |  | **N** | P | 67 | 0.018 | 0.0016 |
| Spike | A | 570 | 0.073 | 0.0020 |  | **NSP6** | V | 149 | 0.018 | 0.0006 |
| Spike | D | 1118 | 0.073 | 0.0019 |  | **NSP14** | N | 129 | 0.018 | 0.0024 |
| Spike | T | 716 | 0.073 | 0.0020 |  | **N** | D | 377 | 0.017 | 0.0009 |
| Spike | S | 982 | 0.073 | 0.0018 |  | **Spike** | L | 5 | 0.017 | 0.0008 |
| NSP3 | A | 890 | 0.072 | 0.0270 |  | **NSP12** | A | 656 | 0.016 | 0.0009 |
| N | S | 194 | 0.061 | 0.0032 |  | **NSP6** | M | 86 | 0.015 | 0.0010 |
| Spike | S | 477 | 0.053 | 0.0019 |  | **NS7a** | T | 14 | 0.015 | 0.0007 |
| N | M | 234 | 0.051 | 0.0018 |  | **NSP2** | V | 381 | 0.015 | 0.0008 |
| NSP6 | L | 37 | 0.048 | 0.0007 |  | **NS3** | R | 122 | 0.014 | 0.0011 |
| NSP12 | V | 776 | 0.046 | 0.0017 |  | **N** | P | 13 | 0.014 | 0.0004 |
| NSP13 | E | 261 | 0.046 | 0.0016 |  | **NSP3** | T | 1189 | 0.013 | 0.0005 |
| NSP12 | A | 185 | 0.046 | 0.0013 |  | **NS3** | K | 75 | 0.013 | 0.0008 |
| NSP4 | M | 324 | 0.045 | 0.0014 |  | **Spike** | D | 215 | 0.013 | 0.0007 |
| NSP13 | K | 218 | 0.045 | 0.0015 |  | **NSP2** | A | 318 | 0.013 | 0.0003 |
| N | A | 376 | 0.045 | 0.0013 |  | **NSP5** | P | 132 | 0.013 | 0.0003 |
| NSP6 | L | 142 | 0.044 | 0.0006 |  | **NSP12** | T | 739 | 0.012 | 0.0014 |
| N | P | 199 | 0.040 | 0.0031 |  | **NSP3** | M | 1788 | 0.012 | 0.0017 |
| NSP13 | H | 290 | 0.038 | 0.0017 |  | **Spike** | D | 80 | 0.012 | 0.0009 |
| Spike | N | 439 | 0.037 | 0.0004 |  | **NSP15** | K | 13 | 0.012 | 0.0002 |
| NSP3 | I | 1683 | 0.037 | 0.0017 |  | **NSP6** | M | 143 | 0.011 | 0.0005 |
| NSP13 | K | 460 | 0.037 | 0.0133 |  | **NS3** | S | 166 | 0.011 | 0.0005 |
| NS3 | G | 172 | 0.036 | 0.0032 |  | **NS3** | H | 182 | 0.011 | 0.0012 |
| NSP2 | T | 85 | 0.033 | 0.0045 |  | **Spike** | D | 1163 | 0.011 | 0.0021 |
| NS7b | S | 5 | 0.032 | 0.0004 |  | **N** | H | 145 | 0.011 | 0.0004 |
| NSP13 | A | 598 | 0.032 | 0.0015 |  | **N** | Q | 9 | 0.011 | 0.0005 |
| NSP3 | A | 1736 | 0.031 | 0.0011 |  | **NSP2** | L | 550 | 0.011 | 0.0034 |
| N | P | 365 | 0.031 | 0.0007 |  | **Spike** | Y | 453 | 0.011 | 0.0030 |
| NSP12 | V | 720 | 0.030 | 0.0018 |  | **NS9b** | S | 6 | 0.011 | 0.0006 |
| Spike | A | 262 | 0.030 | 0.0005 |  | **Spike** | G | 1167 | 0.010 | 0.0022 |
| NSP6 | K | 270 | 0.030 | 0.0009 |  | **NSP15** | R | 207 | 0.010 | 0.0013 |
| NSP9 | M | 101 | 0.030 | 0.0016 |  | **NSP7** | M | 75 | 0.010 | 0.0008 |
| NSP15 | T | 34 | 0.029 | 0.0017 |  | **NSP5** | G | 71 | 0.010 | 0.0007 |
| NS3 | T | 223 | 0.026 | 0.0012 |  | **NS9b** | H | 9 | 0.010 | 0.0005 |
| NSP3 | T | 1363 | 0.025 | 0.0008 |  | **NSP15** | T | 115 | 0.010 | 0.0003 |
| NS3 | V | 202 | 0.023 | 0.0009 |  | **NSP5** | G | 15 | 0.010 | 0.0008 |
| NS8 | S | 24 | 0.022 | 0.0023 |  |  |  |  |  |  |

**Figure S1.** Residue mutation rates for the following SARS-CoV-2 highly conserved proteins: NSP1, NSP2, NSP3, NSP4, NSP5 (Mpro), NSP6, NSP7, NSP8, NSP9, NSP10, NSP11, NSP13, NSP14, NSP15, NSP16, NS3, NS6, NS7a, NS7b, NS8, NS9b, Envelope (E) and Membrane (M).


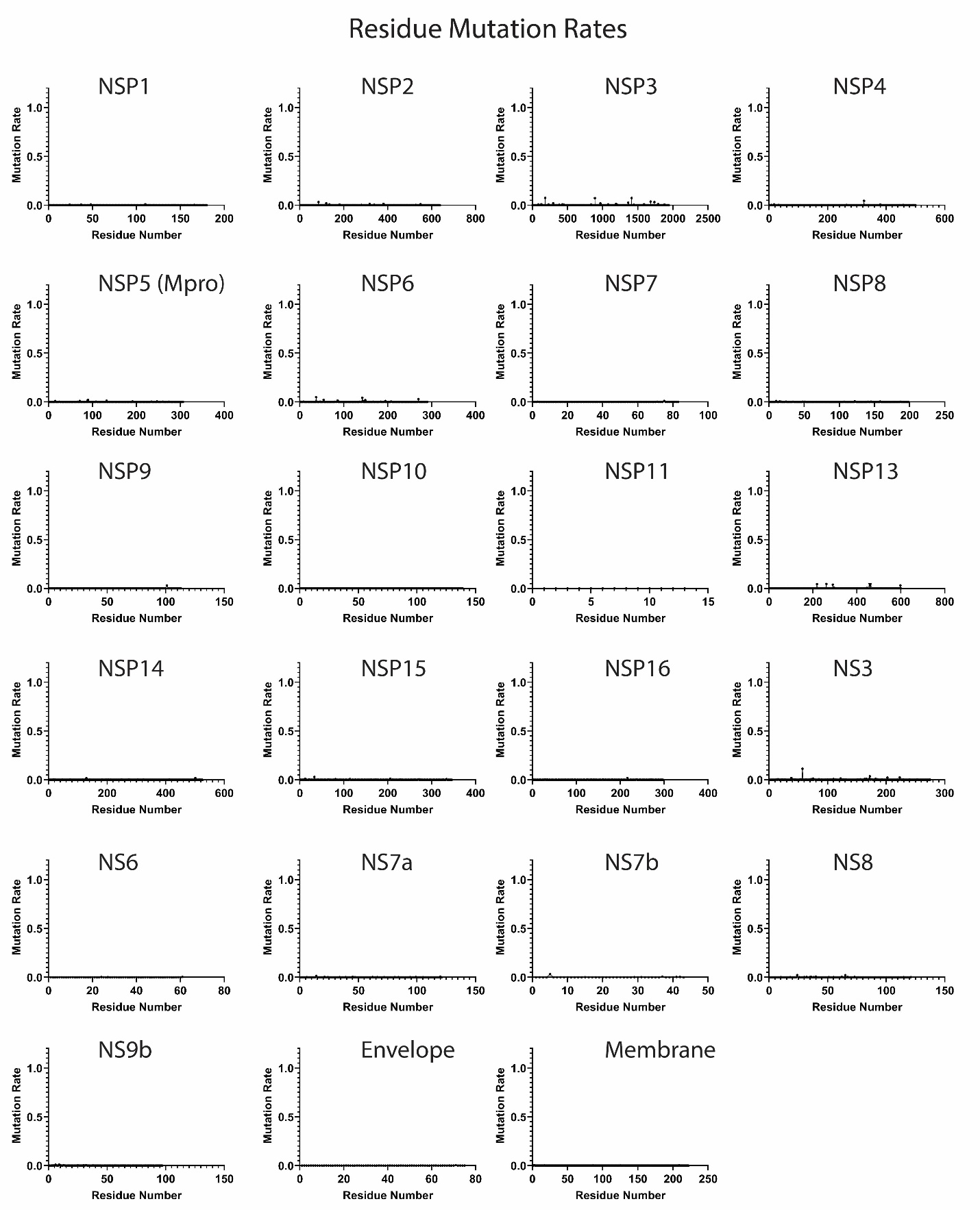

Supplement: Supplementary file 1 [file biology-10-00091-s001.zip › Biology_Suppl_Mat_TableS1_FigS1.docx]
